# The New Territorial Force Regulations

**Published:** 1910-09-10

**Authors:** 


### The Royal Army Medical Corps Section.

### THE NEW TERRITORIAL FORCE REGULATIONS.

It was in July 1908 that there was published a volume of "Regulations for the Territorial Force and for the County Associations," but it was very properly stated that they were of a provisional nature, and to a large extent tentative and experimental, there having been no practical experience in the working of the new military system that they introduced. Now that two years have elapsed since the Territorial Force came into existence the military authorities think that they are in a position to decide definitely on the regulations necessary for it, confirming those that have stood the test of time, and modifying those that have not proved satisfactory. Consequently they have just published a new issue, which is no longer provisional in its character, but takes its place as a permanent standing code or authority, an order by his Majesty giving an official stamp to the contents and making them the sole authority on the matters of which it treats. Like its predecessor it is made up of two parts, one of which deals with the Territorial Force, and the second with the County Associations. There are nineteen appendices, three of which are new. Of the old ones, number eleven has been expanded so as to include rules for the examination and promotion of the N.C.O.s of the R.A.M.C. (T.F.). Lastly, a table of definitions, and another explanatory of abbreviations completes the book.

### The Changes in the Territorial Service.

Many of the changes announced in these 1910 Regulations have been promulgated in the circular memoranda and Army orders published during the last two years, so that they are already known and have been in many cases acted on, but attention is drawn to them by a black line in the margin of the new issue, and its presence on nearly every page indicates how numerous the alterations are. As some of them are due to points raised by County Associations in their administrative work, this fact is indicated by the name of the association and by a marginal number. Several of the modifications and amendments introduced are in connection with the Territorial Medical Service, and to these we propose to draw attention, even though they are already known and been in force.

We are glad to see that the duties of the administrative medical officer of a division are clearly laid down. Not only does he command, but he has entire administrative charge of all the R.A.M.C. (T.F.) units within the divisional area of his division. This ruling removes difficulties that were felt in the early days of the Territorial Force when the position of the A.M.O. to outstanding units such as the mounted brigade field ambulances was not clearly defined. Now they are entirely under him, and a special paragraph gives him charge of their technical training. The same holds good with the medical officers doing duty with the coast defence troops. No increased pay or allowances have been given to the A.M.O. of a division, as we think should have been done, and his travelling expenses have been fixed at £40 per annum. The rule that makes the senior executive officer in a field unit the commandant of the corps at a station should relieve the A.M.O. of a good deal of detail, as this executive officer becomes the channel of communication for all other units. Appendix 8 with its diagram shows very clearly the chain of command, and brings out the grouping of all the medical units under the divisional commander, and consequently under his A.M.O. While on this matter, it may be further noted that Appendix 9 lays down, also diagrammatically, the different channels of communication between the medical units and County Associations. In the case of individual units they communicate in financial matters direct with the County Associations, but, in accordance with paragraph 72 of the Regulations, at centres with more than one unit of R.A.M.C., the senior medical officer of the field unit who is in command at the station will also be the channel of communication between units and higher military authority, as well as between units and County Associations.

### Promotions.

As regards Lieut.-Colonels, it is laid down definitely in the new Regulations that the basis of promotion to that rank is to be by a system of selection, while paragraph 107 sanctions the transfer of medical officers from one unit of the corps to another, or from a unit of the corps to a combatant unit as medical officer. No changes have been made in the conditions of service for promotion of officers, but honorary colonels are to be appointed only for five years. The appointment of sanitary officers to the sanitary branch is dealt with in some new paragraphs, which give the conditions under which they may be enrolled. They are to be borne on a separate list, according to a fixed establishment of twenty lieutenant-colonels, forty majors, and sixty captains. A special concession has been made that they need not be medical men as long as they are conversant with sanitary science. This latter proviso gives an elasticity of selection that may be widespread in its results, and furnishes a large field for recruiting purposes.

### Hospitals.

The Provisional Regulations of 1908 did not contain any paragraphs dealing with the personnel, training, and mobilisation of the general hospitals. Upon all these points definite information is given in the new issue. No alteration has been made in the permanent personnel, which numbers forty-six as previously, but we observe that for the completion of their establishment on mobilisation the general hospitals are to rely on the good services of the British Red Cross Society in their neighbourhood.

Paragraph 121 of the new Regulations is of importance, as it bears on the procedure that takes place on change of command, and it indicates that a considerable amount of responsibility rests on commanding officers of units during their tenure of office, especially in connection with equipment, for it states that before the retirement of a commanding officer is carried out the general officer commanding-in-chief will ascertain that the certificates required in accordance with the Equipment Regulations, Part III., Territorial Force, are in order, and that the County Association is satisfied as regards any association property that has been under his charge and the accounts of such property. Scrutiny of this kind, unless carried out with consideration and fairness, may be a source of great annoyance and of unjust claims.

As regards the N.C.O.s and men Appendix 11 gives, for the first time, very full rules as to the examination and promotion to non-commissioned rank, and as to the appointment of dispensers. They are on the lines of the Regular R.A.M.C. regulations, but with modifications; for instance, the Territorial sergeant is not compelled to qualify as a compounder to get his rank. The four paragraphs devoted to the water-duty section of the R.A.M.C. (T.F.) assign them as an addition to the establishment of the field ambulances. They are to train with them, and to pass through the school of instruction, so that they will get the usual recruit drill laid down for the R.A.M.C. corps, which includes first aid and stretcher drill. They will also have nursing instruction with their respective units, as they are to assist the regimental medical officers when in the field. This meets the want of hospital attendants that has been long felt in connection with the brigade camp hospitals. Regimental officers should note that in paragraph 372 very definite instructions have been laid down for the training of the regimental stretcher-bearers, and that these cannot be evaded.

A perusal of these 1910 regulations brings home to one how closely the Territorial Force is allied to the Regular Army, especially in all the details of pay and allowances, but it is hoped that in the carrying out of them the military authorities will insist on individual departments doing so in a conciliatory and courteous manner. The rough and ready type of Army management is not suited to the civilian soldier, and is quite unnecessary. In fact, we do not think that it is even yet realised by the junior ranks of Army officers how much lies with them in the matter of the success or failure of our Citizen Army.

